# The seafloor from a trait perspective. A comprehensive life history dataset of soft sediment macrozoobenthos

**DOI:** 10.1038/s41597-023-02728-5

**Published:** 2023-11-17

**Authors:** Kasper J. Meijer, Joao Bosco Gusmao, Lisa Bruil, Oscar Franken, Ise A. Grimm, Tjisse van der Heide, Nadia Hijner, Sander J. Holthuijsen, Lisa Hübner, David W. Thieltges, Han Olff, Britas Klemens Eriksson, Laura L. Govers

**Affiliations:** 1 Groningen Institute for Evolutionary Life Sciences (GELIFES), University of Groningen, P.O. Box 11103, 9700 CC Groningen, The Netherlands; 2 Programa de Pós-Graduação em Geoquímica: Petróleo e Meio Ambiente (POSPETRO) Institute of Geosciences, Federal University of Bahia (IGEO, UFBA), Salvador, Bahia Brazil; 3 Environmental and Marine Biology, Åbo Akademi University, 20500 Turku, Finland; 4 NIOZ Royal Netherlands Institute for Sea Research, Department of Coastal Systems, P.O. Box 59, 1790 AB Den Burg, Texel The Netherlands; 5 Rijkswaterstaat Noord Nederland, P.O. Box 2232, 3500 GE Utrecht, the Netherlands

**Keywords:** Macroecology, Community ecology, Conservation biology, Ecosystem ecology

## Abstract

Biological trait analysis (BTA) is a valuable tool for evaluating changes in community diversity and its link to ecosystem processes as well as environmental and anthropogenic perturbations. Trait-based analytical techniques like BTA rely on standardised datasets of species traits. However, there are currently only a limited number of datasets available for marine macrobenthos that contain trait data across multiple taxonomic groups. Here, we present an open-access dataset of 16 traits for 235 macrozoobenthic species recorded throughout multiple sampling campaigns of the Dutch Wadden Sea; a dynamic soft bottom system where humans have long played a substantial role in shaping the coastal environment. The trait categories included in this dataset cover a variety of life history strategies that are tightly linked to ecosystem functioning and the resilience of communities to (anthropogenic) perturbations and can advance our understanding of environmental changes and human impacts on the functioning of soft bottom systems.

## Background & Summary

Traditionally, changes in species assemblages are examined to understand the response of communities to underlying environmental conditions^1^. However, biogeographical variation in species distributions causes regional variation in the species pool. Effects on species communities caused by environmental shifts can then be obscured when studying systems over large geographical gradients as the spatial shifts in species assemblages can form a major confounding factor^2^. These complications can be overcome by adopting functional traits in addition to the traditional species-based approach^2–4^. The functional trait approach is centred around the environmental filtering and habitat templet concepts^2,5,6^. Environmental conditions select for species with certain characteristics, creating species assemblages with similar functional traits^6,7^. Across large spatial scales, similar abiotic conditions can host diverse species communities which are still functionally similar. Thus, functional traits can help to understand why different taxonomic entities group together in specific habitats, whereas it is more complex to understand the ecological mechanism behind the relationship between species distributions and habitat characteristics purely based on taxonomy alone^8–10^. As such, a functional trait approach allows for the generalisation of community responses to the environment which makes it an ideal analytical approach in studies on ecosystem functioning as well as large scale human impact studies.

Functional traits revolve around phenotypic characteristics of species that determine both their response to environmental stressors^11^ and their effects on ecological processes^12^. These functional traits synergistically determine a species’ fitness by affecting growth, survival, and reproduction^13^. Only species with a set type of traits can be successful under certain environmental conditions following the habitat templet concept^14,15^. With this knowledge, a functional trait approach can identify communities exposed to, and/or sensitive to, disturbances^16^. Additionally, the type of traits chosen to include in the analysis determines the functional aspects of the environment that can be evaluated. Changes in the occurrence of these traits can then be translated into changes or loss of ecosystem services^2,17^. For example, the potential for carbon sequestration in marine soft sediment systems is dependent on bioturbation activity of macrobenthic species which together with body size and mobility determines aeration of the soil among many other factors^2^. Multi-trait-based approaches then allow for more holistic assessments of ecosystem functioning.

The assessment of ecosystem functioning rapidly improves in quality with the inclusion of more traits^3^. However, compiling information of many traits for numerous species is time consuming, especially when the aim is to assess species communities over large geographical extents where species numbers can quickly accumulate. Information on traits is lacking for many species or only accessible through grey literature often in languages other than English. Explicit selection of traits that are expected to be relevant to specific research questions, e.g. bioturbation, body size, and living depth for nutrient cycling^18^, may reduce the time required to compile comprehensive trait datasets. On the other hand, open access availability of already compiled datasets would accelerate the application of trait approaches to current questions^19^. The access to such datasets can then more rapidly give insights in the change of ecosystem functioning, especially in the face of biodiversity loss^19^. In addition, such datasets can continuously be expanded with new trait categories depending on the questions that need to be answered, as well as new species to expand the geographical boundaries for which the analysis can be performed. The availability of dynamic open access trait datasets can therefore facilitate the generalisation of species and community responses to changing conditions and anthropogenic influences^20^.

The south-eastern range of the North Sea from the Netherlands to Denmark is characterised by a system of barrier islands that define a large intertidal soft-bottom ecosystem called the Wadden Sea. The macrozoobenthic community in the Wadden Sea is a pivotal part of many ecosystem functions and services^21,22^. For example, macrozoobenthos is involved in many biogeochemical pathways and nutrient fluxes^23^, but also provides important feeding grounds for commercially important fish species^24^. Thus, changes in macrozoobenthic communities due to environmental changes has implications for ecosystem functioning, which can also affect higher trophic levels such as predatory fish and bird species. The Wadden Sea is a key area for millions of migratory waders as well as an important area for many migratory fish species, that both depend on the productive macrobenthic communities as an important food source^25–27^. This emphasises the need to understand community dynamics and functioning of the macrozoobenthic community in this system.

While several datasets on macrozoobenthic traits are available^28–30^, a comprehensive dataset on functional traits for the macrozoobenthic species found in the Dutch Wadden Sea is not available despite the large monitoring programmes focusing on the macrozoobenthic communities in the Dutch Wadden Sea^30–32^. The beginning of such a trait dataset was compiled by Gusmao *et al*.^16^, including many of the intertidal species found in the Dutch Wadden Sea^30–32^. This dataset contains mostly functional trait categories related to the resistance of species to perturbations, such as living depth and body size^8,29,33^. However, traits related to recovery, such as reproductive frequency and mobility, are equally important to understand the recovery of communities after a disturbance as well as the (re)colonisation potential of many species^29^. Therefore, we here present an expanded dataset adapted from Gusmao *et al*.^16^, containing 235 taxonomic units found in multiple sampling campaigns over the entire Dutch Wadden Sea^30–32^. This new comprehensive dataset complements the intertidal species list and also includes sublittoral species. In addition, it provides 10 new functional traits regarding the response of macrozoobenthic communities to disturbances taking into account both resistance and recovery related functional traits^8,29,33^. Of the 235 taxa occurring in this dataset, only 33 taxa (14%) overlap with the dataset of Clare *et al*.^28^, and 94 taxa (40%) occur in the dataset of Beauchard *et al*.^29^ that is included in the “Btrait” R package^34^. Of all our included taxa, the Marine Species Traits portal^35^ only contains information on body size for 185 (79%) taxa, on feeding mode for 31 (31%) taxa, and on larval development location for 2 (1%) taxa. It also adds six traits not included in the dataset of Clare *et al*.^28^, and six traits not included in the dataset of Beauchard *et al*.^29,34^.

Given that many species included in this dataset are also present in adjacent areas such as the North Sea, it is applicable to a wider range of study systems. Applicability of this dataset is further greatly enhanced by publishing it in a dynamic form that can be periodically updated to include new species and trait categories. This is essential to ensure its long-term applicability as new information on biological traits and ecological functioning can be added as they are published as well as expand to other areas. Having such a comprehensive trait dataset readily available will reduce the time lag between data-collection and trait-based analyses to study impacts of environmental changes and human impacts. Ultimately, this can facilitate the translation of these findings into management actions.

## Methods

### Study area

**Fig. 1:**
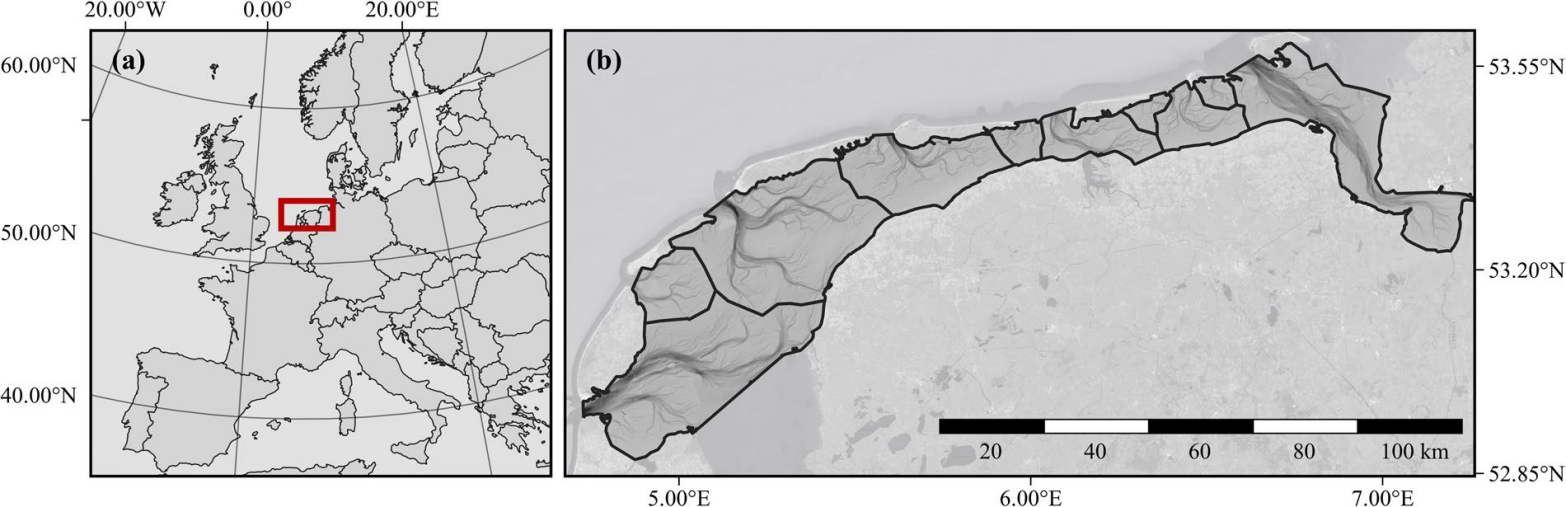
Map of study area. (**a**) Map showing north-west Europe, highlighting the Dutch Wadden Sea in red; (**b**) closeup of the Dutch Wadden Sea with underlying bathymetric map and tidal basins indicated by black lines.

The Wadden Sea (Fig. 1) is one of the largest and most important intertidal ecosystems worldwide^36,37^. Its unique geomorphological and ecological processes as well as its characteristic biodiversity and sheer abundance of protected species has led to the designation as UNESCO World Heritage site for the Dutch and German areas of the Wadden Sea in 2009^38^ and the Danish area in 2014. Large parts of its ecological functioning is determined by macrozoobenthic communities^39^. Likewise, these macrozoobenthic communities are of key importance for higher trophic levels^38^.

### Dataset

The trait dataset was constructed containing 16 life history traits (Table 1), adapted from Gusmao *et al*.^16^, and extended for all taxa found within a large subtidal sampling campaign in 2019^32^. The initial dataset contained information on 71 taxonomic units for bioturbation, living depth, adult body size, feeding mode, longevity, and adult movement. The current dataset has been extended to include 235 taxonomic units, of which 187 on species level, and 10 new trait categories: age of sexual maturation, reproductive frequency, fecundity, living habitat, reproductive mode, larval development location, skeleton, reproductive season, offspring size, and offspring type. Each trait is divided into several modalities that reflect the range of possible attributes a taxa can display. Information for each taxonomic unit was collected from research articles^23,40–527^, textbooks^62,251,528–597^, and existing datasets and species reviews^29,598–706^ to compile a holistic trait dataset for macrozoobenthic taxa occurring in the Dutch Wadden Sea. Primary literature was given preference when available, and expert judgement was only used when no literature could be found. Taxa were scored for each modality using fuzzy coding^707^ with a score ranging from 0 to 3. Here, a score of 0 means no affinity with the scored modality, and 3 indicates absolute affinity with the modality. Hence, when given a score of 3 for a certain modality, the species in question has no known affinity for any of the other modalities within that category. Taxa expressing affinity for multiple modalities can be incorporated by scoring the modality with a 2 or a 1. Multiple scores of 2 would indicate equal affinity for multiple modalities whereas a 1 is a weaker affinity for a certain modality than for others. Fuzzy coding allows for the incorporation of interspecific variation in trait expression^2^ as well as uncertainty in trait expression^708^. In addition, fuzzy coding facilitates a common coding method for both binary classified traits (e.g. bioturbation types) and continuous classifications (e.g. life span)^709^. The 0–3 coding scheme used here is most commonly used across other studies^3,709–711^ and advocated as a standard coding scheme by Degen *et al*.^709^ In case no information on a trait was available for a certain taxa all modalities are scored as a 0. Ultimately, the species-trait combination for which no data was available is effectively not included in the analysis. The fuzzy scores can then be standardized to a score between 0–1 using a min-max normalization to assign individual weights to modalities within all traits and keeping the overall weight of all traits equal regardless of the number of modalities^707,708^. The predictive power of the trait dataset only increases with the inclusion of new information on species life history and functional traits.

**Table 1:** Overview of missing data per class.

Class	Adult body size (mm)	Adult living depth (cm)	Adult living habitat	Adult locomotion	Age sexual maturation (y)	Bioturbation type	Fecundity	Feeding Mode	Larval development location	Longevity (y)	Offspring size (μm)	Offspring type	Reproductive frequency	Reproductive mode	Reproductive season	Skeleton	Overall
Anthozoa (5)					2		3						2				7 (9%)
Ascidiacea (2)																	0 (0%)
Asteroidea (2)																	0 (0%)
Bivalvia (27)							3				1		2		1		7 (2%)
Clitellata (1)		1									1						2 (13%)
Echinoidea (1)																	0 (0%)
Gastropoda (8)																	0 (0%)
Gymnolaemata (10)	1				4		7			9	3		6		5		35 (22%)
Hydrozoa (7)					1		2			1			1		1		6 (5%)
Malacostraca (63)		1			3		5	1		3	4		7		6		30 (3%)
Nematoda (1)		1															1 (6%)
Nemertea (2)		1									1				1		3 (9%)
Ophiuroidea (3)																	0 (0%)
Polychaeta (96)		8	1	1	4		3			4	2		14		14		51 (3%)
Polyplacophora (1)																	0 (0%)
Pycnogonida (1)													1				1 (6%)
Thecostraca (5)																	0 (0%)
Total (235)	1 (0.4%)	12 (5%)	1 (0.4%)	1 (0.4%)	14 (6%)		23 (10%)	1 (0.4%)		17 (7%)	12 (5%)		33 (14%)				143 (4%)

Taxa included in the dataset have been categorized by taxonomical class except for Nematoda and Nemertea which are on phylum level. Numbers behind each class represent the number of taxa within that class included in the dataset. For each trait the number of taxa within that class with missing data is indicated. Lastly, the overall number of missing data per trait and class is shown.

The trait dataset was compiled by several contributors. To assure validity of the dataset, the entire dataset was thoroughly checked by two of the co-authors. Irregularities and debatable scores were then checked and discussed with specialists.

A dynamic dataset was created in R-Studio, R-version 4.2.2^712^. using the ‘shiny’^713^, ‘shinydashboard’^714^, and ‘DT’^715^ packages.

### Definition of traits

In the following section we provide a description and the rationale behind each functional trait category. For an overview of each category and a description of each of its modalities see Supplementary Table 1.

#### Bioturbation mode

Bioturbation is the reworking of soil and sediment through animal and plant activity^716–719^. Bioturbating activity has an important effect on many ecosystem functions, such as sediment stabilisation, nutrient cycling, and carbon sequestration^2,39,720^. Modalities within bioturbation have been adapted from Gusmao *et al*.^16^, and include epifauna, surficial modifier, upward conveyor, downward conveyor, biodiffuser, and regenerator (Supplementary Table 1).

#### Adult living depth (cm)

The living depth indicates the depth range within the sediment that different macrozoobenthic taxa reside in. Species with deeper depth ranges have a greater chance of survival as they are less vulnerable to bottom disturbances^29^. Additionally, bioturbation mode and living depth interact to determine the depth penetration of oxygenated water and thus have different effects on nutrient cycling^18^. Modalities within living depth have been adapted from Gusmao *et al*.^16^, and include surface, >0 and ≤3 cm, >3 and ≤8 cm, >8 and ≤15 cm, >15 and ≤25 cm, and >25 cm (Supplementary Table 1).

#### Adult body size (mm)

Body size correlates with many functional factors among which are food web structure, trophic level, and energy transfer^721,722^. In addition, adult body size is an indicator for the susceptibility of mechanical or physical disturbances. Smaller sized individuals can more easily escape bottom impact^29,723^ and larger sized species are generally more heavily impacted by bottom disturbance due to a higher mortality rate but also a slower recovery rate^723^. Modalities within body size have been adapted from Gusmao *et al*.^16^, and include ≤5 mm, >5 and ≤10 mm, >10 and ≤20 mm, >20 and ≤40 mm, >40 and ≤80 mm, >80 and ≤160 mm, and >160 mm (Supplementary Table 1)

#### Feeding mode

Feeding mode is an important indicator of the functional role of species within the food web as well as the trophic level of a species within the food web (e.g. detritivore, herbivore or predator)^721^. Modalities within feeding mode have been adapted from Gusmao *et al*.^16^, and include deposit-feeder, suspension-feeder, grazer, opportunist or scavenger, and predator (Supplementary Table 1).

#### Longevity (y)

Longevity is a good indicator of population stability over time but also the dispersal potential in combination with mobility as longer living species simply have more time to colonise new areas^721^. In addition, longevity is generally considered a good proxy for life-history strategy as longer living species are usually associated with long generation times and thus highly sensitive to disturbance^724^. Modalities within longevity have been adapted from Gusmao *et al*.^16^, and include the categories: ≤1 year, >1 and ≤3 years, >3 and ≤6 years, >6 and ≤10 years, and >10 years (Supplementary Table 1)

#### Age of sexual maturation (y)

The age of sexual maturation determines the generation time of a population and has implications for the recovery of populations after a disturbance^721^. Modalities within this functional trait have been categorised into ≤1 year, >1 and ≤2 years, >2 and ≤5 years, >5 and ≤10 years, and >10 years (Supplementary Table 1)

#### Reproductive frequency

The frequency with which species reproduce in combination with the abundance of species is important in determining the speed with which a population can recover after a disturbance^721^. Reproductive frequency has been categorised into continuous / ≥ 2x per year, annual 1x,biennial, and semelparous (Supplementary Table 1).

#### Fecundity

The fecundity of taxa in combination with reproductive mode affect the recoverability of taxa after a disturbance. Higher fecundity implies higher probability of young individuals with each reproductive event, decreasing the time it takes to restore the population to a pre-disturbed state^721^. Here, fecundity is defined as the reproductive output per reproductive event in the unit of the type of offspring released. Modalities within fecundity have been categorised into ≥1 and ≤50, >50 and ≤500, >500 and ≤2.500, >2.500 and ≤10.000, >10.000 and ≤20.000, >20.000 and ≤100.000, and >100.000 offspring (Supplementary Table 1)

#### Adult locomotion

The locomotion of macrozoobenthic organisms is an indicator for the recolonisation potential of taxa after a disturbance^29^. Modalities within adult locomotion have been adapted from Gusmao *et al*.^16^, and include the categories: Sessile, swim/float, crawl/walk, and burrow/tube (Supplementary Table 1).

#### Adult living habitat

The living habitat of species is an indicator for the susceptibility of species to disturbances and their dependencies on other species. Species that are attached or live on other species are often more vulnerable to disturbances due to their protruding nature^725^. Free-living species are more easily able to escape disturbance or recolonise after disturbances. Tube living species have an extra layer of protection through the formation of external structures^726^. The modalities within living habitats are therefore categorised as tube, burrow, free-living, crevice, epi/endo-zoic/phytic, and attached (Supplementary Table 1).

#### Reproductive mode

The mode of reproduction is an indicator for the recovery of populations after disturbance as well as the vulnerability of larva, eggs, or juveniles after release. Parental care through brooding generally increases the survival rate of new individuals whereas broadcasted eggs are generally more vulnerable^727–729^. On the other hand, egg sacs that are deposited on the sediment or attached to structures might be vulnerable to physical disturbances or predation^730^. Modalities within reproductive mode have been categorised as asexual, broadcast, brooder, and benthic (Supplementary Table 1).

#### Larval development location

Different larval development locations have different implications for the recolonisation capabilities of species. Planktonic stages can disperse over larger geographical ranges than larvae that directly develop within the sediment^721^. The modalities within this functional trait are categorised into planktonic, lecithotrophic and benthic/direct (Supplementary Table 1).

#### Skeleton

The presence and type of skeleton can have direct implications for the intensity of direct impact on species as it determines the fragility of species to physical disturbance^29^. Additionally, species with calcareous skeletons can be affected by ocean acidification^721^ which can have ramifications for resource use and energy transfer and physiological costs for these species may increase^731,732^. Skeleton has been categorised in the modalities soft, calcified and chitinous (Supplementary Table 1).

#### Reproductive season

Reproductive season can determine recovery potential after seasonal disturbances^733^ and can have implications for management actions such as temporal closures. Reproductive season has been categorised into: Winter, spring, summer, and autumn (Supplementary Table 1).

#### Offspring size (µm)

Offspring size is an indicator of development speed and thus of recovery after disturbances^29,727,728^. Offspring size has been categorised into: ≤100 µm, >100 and ≤500 µm, >500 and ≤1500 µm, and >1500 µm (Supplementary Table 1).

#### Offspring type

The type of offspring released affects the vulnerability of the earliest life stage to environmental and biotic conditions as well as determines the development speed of new individuals^29^. Broadcasted eggs are more susceptible to planktotrophy than are individuals immediately released as larvae or juveniles^727,728^. Modalities within offspring type have been categorised into: Juvenile, larva, and egg (Supplementary Table 1).

### Statistical analysis

All analyses were conducted in R-Studio, R-version 4.2.2^712^. First, the trait space for all taxa occurring in the dataset was investigated. Trait modality scores were standardised to a value between 0 and 1 where the row sum for each trait equals 1 for every taxa using the ‘ade4’ package^734^. We then evaluated the missingness of data per taxonomic class. The combination of different trait modalities and the trait space was then investigated to identify any impossible combinations. The number of occurrences of each possible combination was first summed. The relative occurrence of each trait modality combination was then determined by dividing the number of occurrences by the number of taxa included in the dataset.

## Data Records

A static version of the dataset as used in the preparation of this paper can be found in the dataverseNL (DANS) repository through 10.34894/Z43J6I^735^. In addition, a dynamic version is hosted at the University of Groningen and can be accessed via marinetraits.web.rug.nl. Here, periodic updates will be uploaded as new traits and/or species are added, and older versions can be retrieved for reproducibility. The dataset can be easily downloaded from here as either a csv or excel file. The dataset consists out of three data sheets: *Metadata*, *Traits*, and *References*

### Metadata

The metadata sheet contains information on the version of the dataset as well as a descriptor of all included trait categories as well as modalities (Supplementary Table 1).

### Traits

The Traits data sheet contains all trait information on the different taxonomic groups. The first two columns give the latest scientific name (reference date: April 28th 2023), as well as the AphiaID of the taxa which can be linked back to the WoRMS database^736^ for the latest taxonomic information. Each subsequent column is a modality belonging to a functional trait (Supplementary Table 1). The modalities have values ranging between 0 and 3 based on fuzzy coding^707^ and the reference column contains numbers linking to the References sheet and is used to refer to literature or other existing datasets used to construct the scores.

### References

The References data sheet contains three columns. The first column is the reference id which is the unique numeric identifier used in the Reference column of the Traits data sheet. The second column is the reference itself as well as certain choices made when compiling the dataset. For example, in some cases, information on species level was missing in which case genus level information was used, this is then indicated through a reference id. Reference id’s for these choices are indicated by letters, whereas other references are numbered. Letters referring to choices based on similar species are always followed by reference id’s relating to the literature on which decisions were made. For higher level taxonomic units, scores were based on species in the dataset belonging to that group and indicated with the letter “p”. Finally, the last column contains any permanent identifiers related to the reference.

## Technical Validation

In total, there were 143 (4%) cases for which information on traits could not be compiled out of 3760 species-trait combinations. This was most often the case for reproductive frequency with 33 cases of missing information (Table 1). Reproductive season had 28 cases of missing information, fecundity had 23 cases of missing information, longevity had 17 cases of missing information, age of sexual maturation had 14 cases of missing information, adult living depth and offspring size had 12 cases of missing information, and adult body size, adult living habitat, adult locomotion, and feeding mode had 1 case of missing information (Table 1). Bioturbation type, larval development location, offspring type, reproductive mode, and skeleton had no cases of missing information (Table 1). Relatively, the Gymnolaemata had the most cases of missing data, followed by Clitellata (Table 1). These classes are also indicated by Tyler *et al*.^737^ as two of the classes with the largest lack of knowledge on functional traits. Most taxa included in the dataset were included within the Polychaeta, Malacostraca, and Bivalvia (Table 1) which absolutely had the highest numbers of missing data (Table 1) but in relative terms only little (3%, 3%, and 2% respectively).

**Fig. 2:**
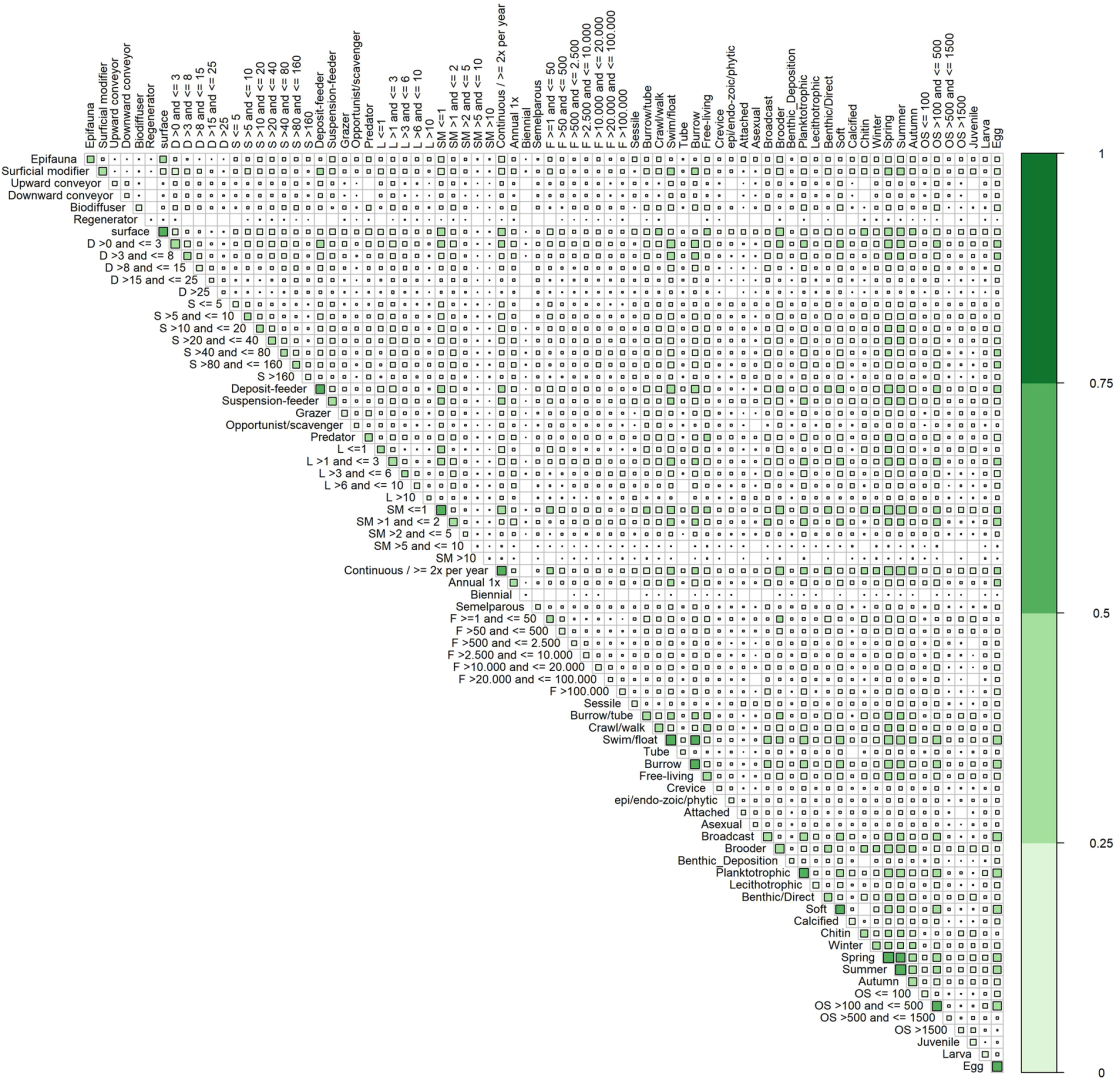
Overview matrix of the trait space. The matrix indicates the presence (green) of combinations between modalities within taxa contained in the dataset. The colour and size represent the relative occurrence of each trait combination within the dataset, with 1 relating to the trait combination present in all taxa included in the dataset. A white square indicates that the specific combination of modalities was not found among the taxa in the dataset.

Out of the 2849 possible modality pairings, 2757 occur in the dataset (Fig. 2). The bioturbation type regenerator, and biennial reproductive frequency were found in the least number of pairings (49 and 46 out of 75 possible pairings respectively) (Fig. 2). Tube living taxa were never associated with high longevity or late sexual maturation (Fig. 2). Predators, chitinous, and epi/endo-zoic/phytic taxa never paired with either upward- or downward conveying bioturbation (Fig. 2). The highest number of occurrences was found for the pairing summer and spring seasonal reproduction (131 out of 235 taxa), followed by swim/float motility and burrowing living habitat (123 out of 235 taxa), and swim float motility and spring seasonal reproduction (114 out of 235 taxa) (Fig. 2). 129 out of 2849 possible modality pairings occurred only in one taxa in the dataset, 176 out 2849 possible pairings only occurred in 2 taxa, and 113 pairings in only 3 taxa. Several impossible combinations of modalities can be found in the dataset. For example, the few occurring combinations of longevity modalities that are lower than the sexual maturation (Fig. 2). This can be attributed to the high intraspecific variability within higher order taxa (e.g. Bivalvia).

## Usage Notes

To build onto this dataset, it is possible for updated versions to be published through the dynamic version of the dataset marinetraits.web.rug.nl. New information, new trait groups or new species can be added to the dataset by sending this information including references to the corresponding author. New information will then be included in the next periodic update of the dataset. Information on the dynamic version of the dataset is liable to change, though older versions of the dataset will remain available through the platform. Note that the data descriptor was peer reviewed in 2023 based on the data available on the platform at the time. This data can be found on the static repository^735^.

Trait based measures can be sensitive to the amount of missing data^738^. Recent advances have been made in methods to impute missing trait information combining phylogenetic information and structural equation modelling^739^. Other imputation methods have been used to fill out missing trait data and can be employed to completely fill out a dataset. Still, each method comes with its own biases and is dependent on the predictive power of the available data and do not always perform better than datasets with missing information^740^. Therefore, no imputation methods have been applied here to fill out missing data and leave this to the user to make sound decisions in the methods employed.

For an overview of imputation methods and their performance in imputing missing trait data see^740^. For review of statistical methods using multiple functional traits see^8^ and^741^.

Utilizing this dataset in combination with other published functional trait datasets^28,34,35^ will greatly enhance the taxonomy covered, expanding the use of functional trait analyses to other regions.

Many sources have been used in the compilation of this dataset, including secondary literature and compiled trait datasets or reviews. Interpretation of trait data may differ on dataset level depending on literature included and the region and can cause variation among datasets. Likewise, some trait information is only available through secondary literature and might not always be quality controlled. Nevertheless, when no primary literature is available, including secondary literature as source of information is preferably better than recording no data for a certain trait. Users are therefore encouraged to check the used literature for applicability of their own analysis and include individual references to acknowledge the original data.

### Supplementary information

Supplementary information to: The seafloor from a trait perspective. A comprehensive life history dataset of soft sediment macrozoobenthos.

## Data Availability

Code used to produce the graphs is available in the dataverseNL (DANS) repository alongside the static version of the dataset through 10.34894/Z43J6I. Figures and analysis were done using Rstudio version 2023.03.0^742^ and R version 4.2.2^712^.
