# Supplementary material for: The seafloor from a trait perspective. A comprehensive life history dataset of soft sediment macrozoobenthos
Source: Sci Data. 2023 Nov 17;10:808. doi: 10.1038/s41597-023-02728-5 (PMC10656422; doi:10.1038/s41597-023-02728-5)
Supplement: Supplementary file 1 — Supplementary information to: The seafloor from a trait perspective. A comprehensive life history dataset of soft sediment macrozoobenthos. [file 41597_2023_2728_MOESM1_ESM.docx]

**Supplementary information to:**

**The seafloor from a trait perspective. A comprehensive life history dataset of soft sediment macrozoobenthos.**

**Supplementary Table 1** Overview of traits and modalities included in the trait dataset.

| **Trait** | **Modality** | **Description** |
| --- | --- | --- |
| Bioturbation type | Epifauna | No bioturbating activity due to epifaunal living environment |
|  | Surficial modifier | Any invertebrate whose sediment reworking activity is restricted to the uppermost (~1 cm) sediment layers (Solan et al 2004) |
|  | Upward conveyor | Head-down oriented fauna which causes active sediment movement from depth to the surface (François et al. 1997) |
|  | Downward conveyor | Head-up oriented fauna which causes active sediment movement from the surface to depth through their gut (François et al. 1997) |
|  | Biodiffuser | Fauna that randomly moves sediment over short distances causing diffusive mixing (François et al. 1997) |
|  | Regenerator | An invertebrate that transfers sediment at depth to the surface where it is washed away and replaced by the sediment of surficial signature (Gardner et al., 1987) |
| Adult living depth (cm) | surface | Surface living species |
|  | >0 and ≤ 3 | Species living between 0-3 cm depth in the sediment |
|  | >3 and ≤ 8 | Species living between 3-8 cm depth in the sediment |
|  | >8 and ≤ 15 | Species living between 8-15 cm depth in the sediment |
|  | >15 and ≤ 25 | Species living between 15-25 cm depth in the sediment |
|  | >25 | Species living deeper than 25 cm in the sediment |
| Adult body size (mm) | ≤ 5 | Body size smaller then 5 mm |
|  | >5 and ≤ 10 | Body size between 5-10 mm |
|  | >10 and ≤ 20 | Body size between 10-20 mm |
|  | >20 and ≤ 40 | Body size between 20-40 mm |
|  | >40 and ≤ 80 | Body size between 40-80 mm |
|  | >80 and ≤ 160 | Body size between 80-160 mm |
|  | >160 | Body size larger than 160 mm |
| Feeding Mode | Deposit-feeder | Taxa that forage along the surface and ingest soft parts of the sediment and so digesting and assimilating organic matter |
|  | Suspension-feeder | Taxa that obtain food by filtering particles from the water column |
|  | Grazer | Taxa that graze on plants and algae |
|  | Opportunist/scavenger | Opportunistically feeding taxa |
|  | Predator | Predatory taxa |
| Longevity (y) | ≤1 | Taxa that live less than 1 year |
|  | >1 and ≤ 3 | Taxa that live between 1-3 years |
|  | >3 and ≤ 6 | Taxa that live between 3-6 years |
|  | >6 and ≤ 10 | Taxa that live between 6-10 years |
|  | > 10 | Taxa that live longer than 10 years |
| Age of sexual maturation (y) | ≤1 | Sexually mature within 1 year |
|  | >1 and ≤ 2 | Sexually mature between 1-2 years |
|  | >2 and ≤ 5 | Sexually mature between 2-5 years |
|  | >5 and ≤ 10 | Sexually mature between 5-10 years |
|  | >10 | Sexually mature after 10 years |
| Reproductive frequency | Continuous / >= 2x per year | Two or more reproductive events per year |
|  | Annual 1x | One reproductive event per year |
|  | Biennial | Reproduces every other year |
|  | Semelparous | Reproduces once in a lifetime |
| Fecundity | ≥1 and ≤ 50 | Reproductive output of 1-50 offspring over its lifetime |
|  | >50 and ≤ 500 | Reproductive output of 50-500 offspring over its lifetime |
|  | >500 and ≤ 2.500 | Reproductive output of 500-2.500 offspring over its lifetime |
|  | >2.500 and ≤ 10.000 | Reproductive output of 2.500-10.000 offspring over its lifetime |
|  | >10.000 and ≤ 20.000 | Reproductive output of 10.000-20.000 offspring over its lifetime |
|  | >20.000 and ≤ 100.000 | Reproductive output of 20.000-100.000 offspring over its lifetime |
|  | >100.000 | Reproductive output of more than 100.000 offspring over its lifetime |
| Mobility | Sessile | Taxa that are sessile and do not move |
|  | Swim/float | Taxa that can move around by swimming or floating through the water column |
|  | Crawl/walk | Taxa that move around by crawling or walking |
|  | Burrow/tube | Taxa that move around by burrowing through the sediment or by creating tubes |
| Adult living habitat | Tube | Taxa that live in tubes |
|  | Burrow | Taxa that burrow through the sediment |
|  | Free-living | Taxa that live freely |
|  | Crevice | Taxa that live in between crevices |
|  | epi/endo-zoic/phytic | Taxa that are attached to other living organisms |
|  | Attached | Taxa that are attached to hard substrates |
| Reproductive mode | Asexual | Taxa that reproduce asexually |
|  | Broadcast | Taxa that reproduce by releasing eggs and sperm in the water column |
|  | Brooder | Taxa that reproduce by internal or external fertilisation but keep the eggs or larvae within the body, or in the burrow (parental care) or a body cavity until hatching |
|  | Benthic_Deposition | Taxa that produce egg sacs that are attached to a substrate |
| Larval development location | Planktotrophic | Larvae grow up planktonic and must feed on plankton for their development into their juvenile stage |
|  | Lecithotrophic | Planktonic development but initial food source is provided by the yolk from its egg |
|  | Benthic/Direct | No planktonic stage. Taxa develop fully within the sediment or brooded to juvenile |
| Skeleton | Soft | No exoskeleton, soft bodied |
|  | Calcified | Calcified exoskeleton |
|  | Chitin | Chitinous exoskeleton |
| Reproductive season | Winter | Species that reproduce in winter |
|  | Spring | Species that reproduce in spring |
|  | Summer | Species that reproduce in summer |
|  | Autumn | Species that reproduce in winter |
| Offspring size (μm) | ≤ 100 | Offspring smaller than 100 µm |
|  | >100 and ≤ 500 | Offspring between 100-500 µm |
|  | >500 and ≤ 1500 | Offspring between 500-1500 µm |
|  | >1500 | Offspring larger than 1500 µm |
| Offspring type | Juvenile | Offspring is released as juvenile |
|  | Larva | Offspring is released as larva |
|  | Egg | Offspring is released as egg |
